# Investigation on Fracture Behavior of Cementitious Composites Reinforced with Aligned Hooked-End Steel Fibers

**DOI:** 10.3390/ma15020542

**Published:** 2022-01-11

**Authors:** Sujjaid Khan, Longbang Qing, Iftikhar Ahmad, Ru Mu, Mengdi Bi

**Affiliations:** School of Civil and Transportation Engineering, Hebei University of Technology, Tianjin 300401, China; sujjaidkhan93@gmail.com (S.K.); iftiuetced@gmail.com (I.A.); ru_mu@hotmail.com (R.M.); 202111601001@stu.hebut.edu.cn (M.B.)

**Keywords:** steel fiber reinforced cementitious composites, aligned steel fiber concrete, mechanical properties, fracture parameters, fiber orientation efficiency factor

## Abstract

Aligning steel fibers is an effective way to improve the mechanical properties of steel fiber cementitious composites (SFRC). In this study, the magnetic field method was used to prepare the aligned hooked-end steel fiber cementitious composites (ASFRC) and the fracture behavior was investigated. In order to achieve the alignment of steel fibers, the key parameters including the rheology of the mixture and magnetic induction of electromagnetic field were theoretically analyzed. The results showed that, compared with SFRC, the cracking load and the ultimate load of ASFRC were increased about 24–55% and 51–86%, respectively, depending on the fiber addition content. In addition, the flexural tensile strength and residual flexural strength of ASFRC were found to increase up to 105% and 100%, respectively. The orientation of steel fibers also has a significant effect on energy consumption. The fracture energy of ASFRC was 56–70% greater than SFRC and the reinforcement effect of hooked-end steel fiber was higher than straight steel fiber. The fibers in the fracture surface showed that not only was the number of fibers of ASFRC higher than that of SFRC, but also the orientation efficiency factor of ASFRC was superior to SFRC, which explains the improvement of fracture behavior of ASFRC.

## 1. Introduction

During the last four decades, steel fiber reinforced cementitious composites (SFRC) have been extensively investigated and have been known for many developments to overcome the tension weakness common to all types of concrete. The main change caused by adding fibers to a cement mixture is the transformation from a quasi-brittle to a pseudo-ductile material, with relatively less catastrophic failures by bridging the micro-cracks and the macro-cracks [1]. The addition of steel fiber increases the tensile strength of the composites and improves the cracking resistance by taking up internal stresses during loading through their tension resistance. Fibers ensure the stress transfer from the matrix to the fibers, which in turn retard their propagation [2] and provide a good bond existing between the fibers and the hardened cement matrix, particularly for long fibers [3]. Moreover, an increase in the length of steel fiber improves the peak pullout load and corresponding slip between the matrix and the fiber, owing to the increase in the effective bonding area of fibers at crack surfaces [4,5]. For this reason, long fibers can provide a stable post-peak response. The application of SFRC effectively improves the engineering performance of structural and non-structural concrete, including the tensile and flexural strength, high fracture energy, load-bearing capacity, durability, impact resistance, and electrical resistivity [6,7,8,9].

In ordinary steel fiber reinforced cementitious composites (SFRC), steel fibers are randomly dispersed in the matrix, which means that the fibers have random orientation in every direction. However, under certain loading conditions, only a few steel fibers that are aligned with the principle tensile stresses in the structure play a role in reinforcement effect. Steel fibers that do not align parallel to the principal tensile stress may not be able to contribute to the performance of the structural elements. As a result, there will be a waste of steel fibers, which increases the construction cost. Therefore, finding an approach to align the steel fibers with the direction of the principal tensile stress in concrete is important to achieve the highest reinforcing efficiency of the steel fibers. At present, in order to prepare aligned steel fiber reinforced cementitious composites (ASFRC), there are two main methods of aligning steel fibers including magnetic field method and flow induction method. In 1977, Miller and Björklund [10] patented the magnetic field method to align steel fibers. After that, Björn [11] aligned the steel fibers by the use of a magnet device. In 2017, Mu et al. [12] realized the alignment of steel fibers with an electromagnetic device and the fiber orientation efficiency factor was about 0.9. Due to the restriction of the device, this method is mainly suitable for prefabricated elements. In terms of flow induction method, the orientation of steel fibers can be optimized during the flow of mixture. Moon and Kang [13] found that the orientation and distribution of fibers mainly occurred in a short flowing distance. Song [14] found that the effect of casting height on fiber distribution was more significant than flow distance and rheology of mixture. Huang et al. [15] developed a L-shaped device to prepare ultra-high performance concrete with favorable fiber orientation. The results showed that compared with the conventional casting method, the flow induced method contributed to 35% greater fiber orientation and 30–60% flexural strength. Without the restriction of the device, it is more suitable for on-site construction.

At present, the aligned steel fiber reinforced cementitious composites (ASFRC) prepared by magnetic field method was widely investigated. Mu et al. studied the mechanical properties of ASFRC. The results showed that compared with SFRC, the flexural strength of ASFRC was increased by 46–167% [12], the toughness index T_150_ was increased by 109–148% [12], the shear strength was increased about 40% [16], and the fracture energy was increased by 31–79% [17]. Moreover, a magnetic device was developed to align steel fibers from one side surface of the mold which is full of fresh mixture. By this means, the magnetic field method can be used in on-site construction and have a satisfied fiber orientation efficiency factor [18]. Besides, the magnetic field method was used to align tubular healing fibers in order to realize the increase in self-healing capability. The permeability test and repetitive splitting tensile test results showed that the aligned healing fibers performed better than the randomly distributed healing fibers [19].

Compared with straight steel fiber, the hooked-end steel fiber has a potential to further improve the mechanical properties and durability. Abdelrahman et al. [20] found that the flexural strength of specimens with hooked-end steel fibers had 50–77% higher strength than the counterparts with straight steel fibers. Wu et al. [21] found that the compressive strength of concrete containing hooked-end fiber increased by 48% compared with the concrete with the same amount of straight fiber. Zhang [22] and Ruano [23] observed that hooked-end steel fiber had a significant effect on residual flexural strength after exposure to high temperature. Moreover, Fang et al. [24] reported that hooked-end fibers were found to be more effective in restraining autogenous shrinkage compared with straight fibers. Combining with the align steel fiber technology, the reinforcement effect of hooked-end steel fiber can be exerted thoroughly. However, the effect of hooked-end steel fiber on the mechanical property, especially the fractural property of aligned steel fiber reinforced cementitious composites (ASFRC), was not investigated and the gain in fractural property was not clear. Besides, in terms of the magnetic field method, the key parameters for aligning steel fibers have not been determined. Both of these issues are important to resolve in order to adequately understand the preparation and properties of ASFRC.

In this work, the magnetic field method was used to prepare aligned steel fiber reinforced cementitious composites (ASFRC) with hooked-end steel fibers. In order to prepare ASFRC successfully, the key parameters were theoretically analyzed and determined through experiment. Besides, the fractural properties of the ASFRC specimens including their load–deflection curve, load–CMOD curve, flexural strength, and fracture energy were carried out and compared with those of ordinary steel fiber reinforced cementitious composites (SFRC). The reinforcement mechanism was also revealed from the aspect of fiber orientation efficiency factor and fiber number at the cracking surface. The purpose of this study is to contribute to the knowledge of ASFRC in the structural field and to help spread its use.

## 2. Materials and Methods

### 2.1. Materials and Mix Design

In this study, hooked-end steel fibers provided by Zhitai Steel Fiber Manufacturing Co., Ltd. (Tangshan City, China) were used with the following volume contents: 0.8%, 1.2%, and 2.0%. The mix proportions of the cementitious composites are presented in Table 1 and the properties of the hooked-end steel fiber are presented in Table 2. Ordinary steel fiber reinforced cementitious composites (SFRC) and aligned steel fiber reinforced cementitious composites (ASFRC) with different volume content of steel fibers were prepared. Both types of composites were prepared with Portland cement (PC) (Tangshan Jidong Cement Co., Ltd, Tangshan, China) of grade P⋅O 42.5, river sand with a fineness modulus of 2.5 (taken from local river, Tianjin, China), and polycarboxylate superplasticizer (Jiangsu Sobute New Materials Co., Ltd., Nanjing, China). These mix proportions had the same water to cement ratio of 0.36 and sand to cement ratio of 1:2.

### 2.2. Preparation of Aligned Steel Fiber Reinforced Cementitious Composites (ASFRC)

#### 2.2.1. The Alignment Principle of Steel Fiber

It is well known that under the influence of the Earth’s magnetic field, the compass will rotate and line up along the geomagnetic line regardless of the initial direction of the compass. This natural phenomenon provided an inspiration for the alignment of steel fibers in cementitious composites. During the preparation of aligned steel fiber reinforced cementitious composites (ASFRC), the fresh mixture is placed in a uniform magnetic field and the steel fibers are magnetized into small magnetic needles. Both the magnetic force generated by the magnetic field and viscous resistance from the matrix on the steel fiber are torque couples, which drive and impede the rotation of the steel fiber, respectively. If the magnetic moment is greater than the viscous moment, the steel fiber can rotate. The less the impeding moment and the greater the driving moment, the easier the aligning of the steel fibers in the fresh mixture.

#### 2.2.2. Force Analysis of Steel Fiber

During the alignment, the forces subjected to the steel fiber in a fresh cement mortar are: weight of steel fiber (W) or gravity, buoyant force ($F_{b}$), magnetic force ($F_{m}$), and viscous resistance ($F_{d}$) [12]. Among them, the gravity and buoyancy, which do not play a role in rotation of steel fibers, are simplified as concentrated forces acted on the center of gravity of the steel fiber. The magnetic force and viscous resistance on the steel fiber are applied as torque couples, which drive and impede the rotating of the steel fiber, respectively. The viscous resistance mainly depends on the rheology of the mixture and the magnetic force is determined by the magnetic induction intensity [25]. In order to make the steel fiber rotate, a reduction in viscous resistance or an increase in magnetic force can be adopted. However, lower viscous resistance may cause the steel fiber to sink, which leads to the poor distribution of steel fibers and has adverse effects on mechanical properties [26]. Therefore, increasing the magnetic force was adopted in this study.

Based on fluid mechanics, the viscous force can be calculated by using Equation (1) [27]:

(1) $$F_{d}=C_{D}Al_{f}\rho \frac{v^{2}}{2}$$

where: $F_{d}$ is the viscous force (N/m) and $C_{D}$ is the non-dimensional drag coefficient. A is the projected area of the fiber on the plane vertical to the direction of rotation (mm^2^), $l_{f}$ is the length of the fiber (mm), ρ is the density of the mixture (kg/m^3^), and v is the relative speed between fiber and matrix (m/s), simply known as the velocity of fibers.

After ensuring the rheology of the mixture, the viscous force $F_{d}$ can be calculated and the magnetic force $F_{m}$ can be ensured as long as $F_{m}$ > $F_{d}$.

When the fibers are aligned by a solenoid, according to the electromagnetism [28], the distributed magnetic force acting on a steel fiber can be approximated by [29]:

(2) $$F_{m}=\frac{\mu -1}{2\mu_{0}\mu l_{f}}B^{2}A_{f}$$

where $F_{m}$ is the distributed magnetic force acting on a steel fiber (N/m), $A_{f}$ is the cross-section area of the fibers ($m^{2}$), B is the magnetic induction (N/A·m), μ is the steel magnetic permeability, also called the relative permeability, and $\mu_{0}$ is the vacuum permeability ($N/A^{2}$). Usually, $\mu_{0}$ = 4π × 10^−7^ (N/A^2^). Furthermore, according to Equation (2), the magnetic induction can be calculated by:

(3) $$B=\sqrt{\frac{2F_{m}\mu_{0}\mu l_{f}}{A_{f}\left(\mu -1\right)}}$$

According to Ampere’s law, the electrical current of the solenoid required to create a sufficient magnetic induction intensity can be calculated as follows [29]:

(4) $$I=\frac{Bl}{\mu_{0}n}$$

where I is the required current (A), n is the number of solenoid turns, and l is the length of the solenoid (m).

In order to ensure the well-distributed and easily-rotated steel fibers, the plastic viscosity of the mixture was eventually determined as 10–300 Pa·s and the magnetic induction should be in the range of 0.015 × 10^−4^ – 2.0 × 10^−4^ T. The corresponding current of solenoid was determined to be about 5–10 A.

#### 2.2.3. Process of Preparing Aligned Steel Fiber Reinforced Cementitious Composites (ASFRC)

The preparation of fresh mixture containing steel fibers was as follows. Firstly, the cement and the sand were mixed for 30 s in a mixing pot. Secondly, the water containing superplasticizer was poured into the mixing pot and mixed for 60 s. Then, the steel fibers were added into the mortar slowly in order to make the steel fibers disperse in the mortar uniformly. Finally, after finishing the procedure of adding the fibers, the mixture was mixed for another 60 s and the fresh mixture containing steel fibers was prepared successfully.

In the process of aligning steel fibers by magnetic field, a solenoid wrapped by copper wire was used to provide the uniform electromagnetic field. The detailed method of aligning fibers is described in [12]. It should be noted that each type of composite was prepared three specimens in order to make the results more accurate and convincing. Before curing for 28 days in the curing room, the specimens were demolded in 1 day.

### 2.3. Notched Three-Point Bending Fracture Test

The notched three-point bending (TPB) test is a standard test method for SFRC described in the CECE 13: 2009 [30]. For this study, 100 × 100 × 440 mm^3^ prismatic specimens were tested in which the span (S) of the beams was 400. All the specimens were precut in the middle of the beams with a notch of 40 mm depth ($a_{0}$) and 5 mm width. For all specimens, the span to depth ratio (S/d) and the notched to depth ratio ($a_{0}/d$) was taken as 4 and 0.4, respectively. The beam was simply supported with the notched face down clamped with the two clip gauges attached at the knife edge having a capacity of 10 mm and placed 5 mm away on both sides of the notch section. The 5 mm knife-edge thickness was chosen according to [31] as an acceptable error level for the crack mouth opening displacement (CMOD) of the specimen of 10%. Besides, two linear variable differential transformers (LVDTs) were attached on each side of the specimens to measure the mid-span deflection. During the test, the loading speed was controlled at 0.05 mm/min for CMOD less than 0.1 mm and at 0.15 mm/min for CMOD greater than 0.1 mm.

### 2.4. Evaluation of Steel Fiber Orientation

After finishing the fracture test, the specimens were broken into two sections along the crack at the notch. In order to analyze the orientation of steel fibers, the manual fiber-counting method was used to record the angle between the steel fiber and the axis of the specimen according to [32]. The angles were divided into four zones, namely, 0–15°, 15–45°, 45–75°, and 75–90°. According to [33], the fiber orientation efficiency factor $\eta_{\theta }$ of the specimen was calculated by:

(5) $$\eta_{\theta }=\frac{\sum_{1}^{N}cos\theta }{Nl_{f}}=\frac{1}{N}\overset{N}{\underset{1}{\sum }}cos\theta_{i}$$

where $\eta_{\theta }$ is the average orientation efficiency factor of the steel fibers, N is the total number of fibers in the cracked section, and $\theta_{i}$ is the angle between the steel fiber and the axis of the specimen.

## 3. Results and Discussion

### 3.1. Load-Deflection Curves

**Figure 1:**
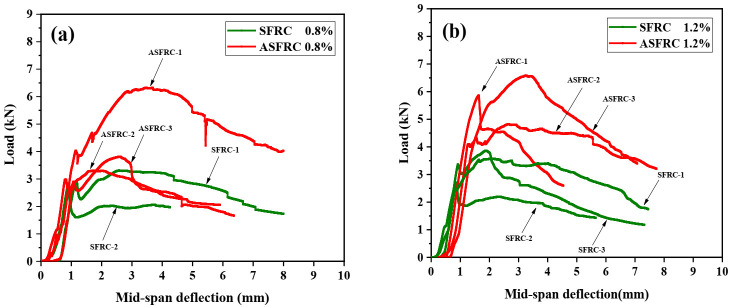

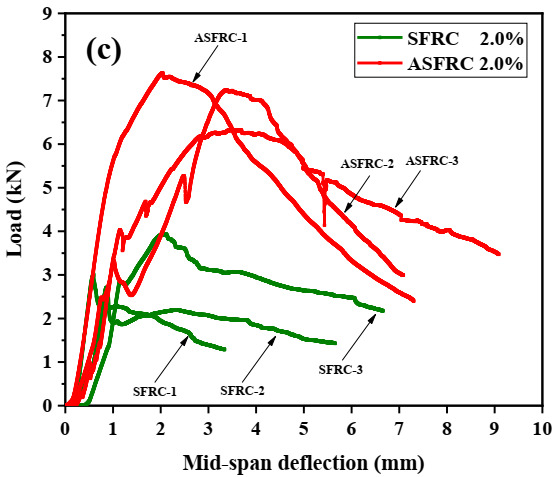
Comparison of load–mid span deflection curves for SFRC and ASFRC with different fiber volume fractions: (**a**) 0.8% of fiber volume fraction, (**b**) 1.2% of fiber volume fraction and (**c**) 2.0% of fiber volume fraction.

Kang [7] and Zhang [34] stated that the effect of the fiber orientation was found to be very small on pre-cracking behavior but significant on post-cracking behavior. The statement was proved by Figure 1. It can be seen in Figure 1 that compared with SFRC, ASFRC had a significant influence on the post-cracking stage of load–deflection curves. Specifically, an elastic and a non-linear phase were found for both the SFRC and ASFRC specimens. After that, the load decreased gradually with further displacement increase. It should be noted that the post-peak behavior was significantly improved by fiber alignment. The alignment of fibers contributed to enlarging the area under the load–deflection curve and improving the energy absorption capacity by fiber bridging effect, leading to an increase in the ultimate load and a more ductile softening behavior during the fracture process for ASFRC. As a result, ASFRC specimens exhibited higher load-bearing capacity and toughness than SFRC, which implies that ASFRC can greatly reduce the brittleness of the specimens than SFRC counterparts.

**Figure 2:**
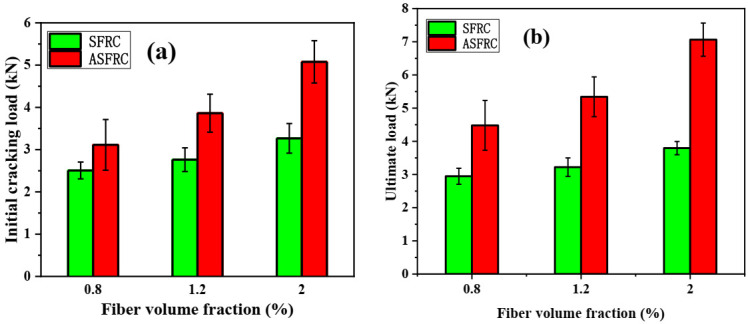
Initial cracking load and ultimate load of ASFRC and SFRC specimens with different fiber volume fractions: (**a**) Initial cracking load and (**b**) Ultimate load.

The initial cracking and ultimate load of ASFRC and SFRC specimens were determined according to load–deflection curves. The initial cracking load is the corresponding load at the knee point of the load–deflection curve at which the curve becomes nonlinear. The initial cracking load and ultimate load are presented in Figure 2. Compared with SFRC, the initial cracking load of ASFRC was increased by 24.15%, 39.80%, and 55.36% and the ultimate load of ASFRC increased by 51.98%, 65.84%, and 86.07% at fiber volume fractions of 0.8%, 1.2%, and 2.0%, respectively. Therefore, the aligned steel fibers were higher than conventional randomly distributed steel fibers in terms of reinforcement efficiency. Mu et al. [12] reported that the ultimate load of ASFRC with the water to cement ratio of 0.36 was increased 46.2%, 89.6%, and 145.6% at fiber volume fractions of 0.8%, 1.2%, and 2.0%, respectively, which was higher than those in this study. This may be because the steel fibers used in the experiments were different. In this study, 25 mm of fiber was used while the length of fiber was 30mm in literature [12]. Due to the increase in the effective bonding area of fibers at crack surfaces, the increase in the length of steel fiber provides advantages in terms of ultimate load and corresponding slip between the matrix and the fiber [4,5].

### 3.2. Load–CMOD Curves

**Figure 3:**
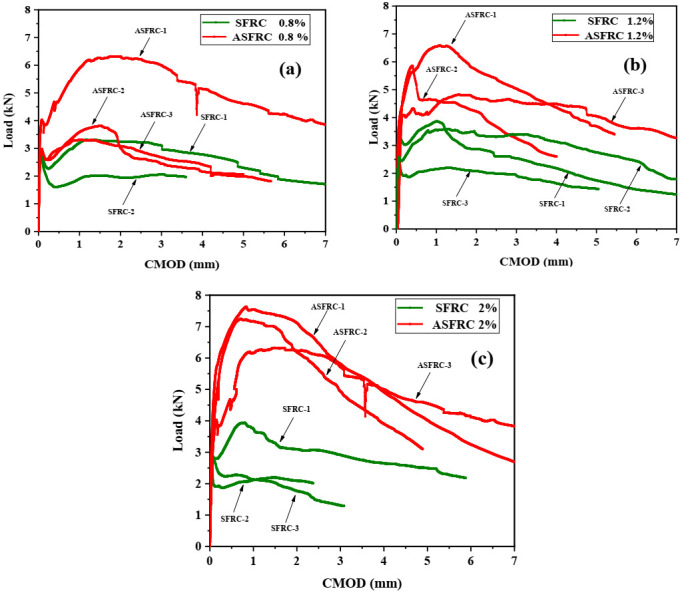
Load-crack mouth opening displacement (CMOD) for ASFR and SFRC at various volume fractions: (**a**) 0.8% of fiber volume fraction, (**b**) 1.2% of fiber volume fraction and (**c**) 2% of fiber volume fraction.

Crack mouth opening displacement (CMOD) is normally used to study the effect of fibers on the post-cracking behavior of cementitious composites. The load–CMOD curves of SFRC and ASFRC are presented in Figure 3. There was a certain difference in fracture characteristics between SFRC and ASFRC. In the initial stage of loading, there was a linear relationship between the CMOD and the load. At this time, the load was mainly maintained by the matrix and the effect of steel fiber can be neglected. After the matrix cracked, the P–CMOD curves of SFRC and ASFRC exhibited nonlinearity. At this stage, the matrix gradually lost its bearing capacity and the load was maintained by steel fibers. With the further improvement of load, the SFRC specimen reached its peak load and then gradually decreased with CMOD. However, not only was the peak load of ASFRC higher than that of SFRC, but also the CMOD corresponding to the peak load of ASFRC was larger than that of SFRC.

Since most of the steel fibers near the cracks of ASFRC specimens can effectively bridge the load, the load transfer capacity is relatively strong. When the CMOD of specimens exceeded about 0.2 mm, the load of the ASFRC specimen was still growing while the load of the SFRC specimen began to fall after the initial crack of the matrix. Specifically, the CMOD corresponding to the ultimate load of SFRC was generally not above 0.2 mm, which was also reported by Wang et al. [35]. However, although the pre-initial crack behavior of ASFRC was similar with SFRC, the ASFRC specimen could still bear higher load until the peak load. Besides, the CMOD corresponding to the ultimate load of ASFRC was significantly higher than that of SFRC. The above phenomenon showed that the aligned steel fibers could significantly enhance the ductility after cracking and improve the energy dissipation capacity. Especially after the peak load, the ductility of ASFRC was improved significantly.

### 3.3. Flexural Strength

#### 3.3.1. Flexural Tensile Strength

The flexural tensile strength $f_{L}$ was calculated according to CECS 13: 2009 [30] using Equation (6):

(6) $$f_{L}=\frac{3F_{L}S}{2bh_{sp}^{2}}$$

where, $F_{L}$ is defined as the maximum load for CMOD ≤ 0.05 mm; b is the width of the beam (mm); and $h_{sp}$ is the effective height of the beam (mm).

**Figure 4:**
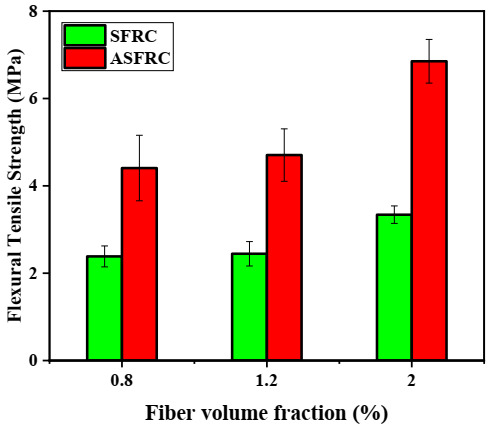
The flexural tensile strength for ASFR and SFRC at various volume fractions.

Analyzing the flexural tensile strength at the limit of proportionality, it can be concluded that the flexural strength of ASFRC is improved after the fiber is aligned to the tensile stress direction compared with SFRC, which was presented in Figure 4. This is because the improved fiber orientation along the principal tensile stress of the specimens enhances the effectiveness of fibers in bridging cracks and resisting cracking [15,36]. Figure 4 shows that compared with SFRC, the flexural strength of ASFRC with a water to cement ratio of 0.36 increased by 17.82%, 29.63%, and 42.12% at fibers volume fractions of 0.8%, 1.2%, and 2.0%, respectively. Similar results were also reported by Huang et al. [36] where the flexural strength of UHPC with orientated steel fibers was 30–50% higher than that of UHPC with random steel fibers. 

#### 3.3.2. Residual Flexure Tensile Strength

The residual flexural strengths $f_{Rj}$ (*j* = 1, 2, 3, 4) were calculated according to EN 14651-2007 [37] using Equation (7).

(7) $$f_{Rj}=\frac{3F_{j}S}{2bh_{sp}^{2}}$$

$F_{j}$ is the load corresponding to CMOD_1_ = 0.5 mm, CMOD_2_ = 1.5 mm, CMOD_3_ = 2.5 mm, and CMOD_4_ = 3.5 mm, respectively.

**Figure 5:**
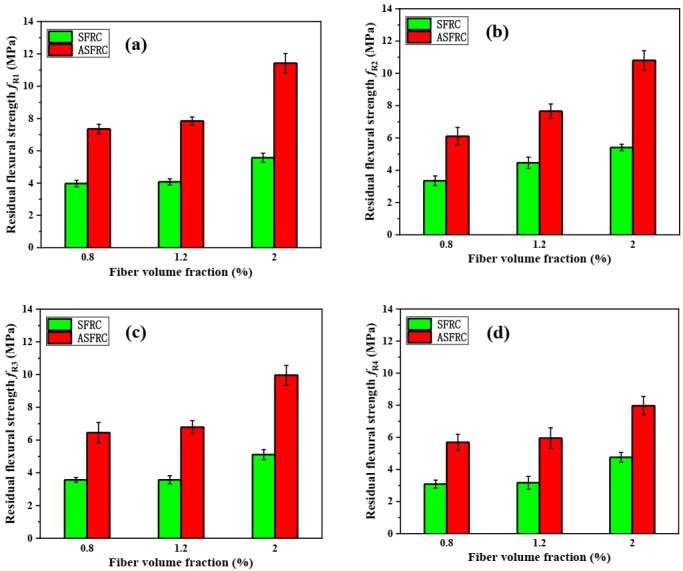
The residual flexural strength of SFRC and ASFRC specimens: (**a**) Residual flexural strength *f*_R1_, (**b**) Residual flexural strength *f*_R2_, (**c**) Residual flexural strength *f*_R3_ and (**d**) Residual flexural strength *f*_R4_.

The average residual flexural strength $f_{R1}$ to $f_{R4}$ for the three SFRC and ASFRC samples are summarized in Figure 5. Compared with SFRC, the residual flexural strength $f_{R1}$ of ASFRC increased by 85%, 92%, and 105%, $f_{R2}\;$ increased by 83%, 72%, and 100%, $f_{R3}$ increased by 81%, 90%, and 95%, while $f_{R4}$ increased by 85%, 88%, and 68% at fiber volume fractions of 0.8%, 1.2%, and 2.0%, evidencing the fiber post-cracking contribution. The gradual increase in fiber content increases the post-cracking performance from CMOD_1_ to CMOD_4_. Besides, it should be noted that the residual flexural strengths $f_{R1}$ and $f_{R4}$ were confirmed with the RILEM TC162 – TDF [38], in which the minimum residual strengths $f_{R1}$ and $f_{R4}$ should be higher than 1.5 MPa and 1 MPa, respectively.

#### 3.3.3. Equivalent Flexure Strength

**Figure 6:**
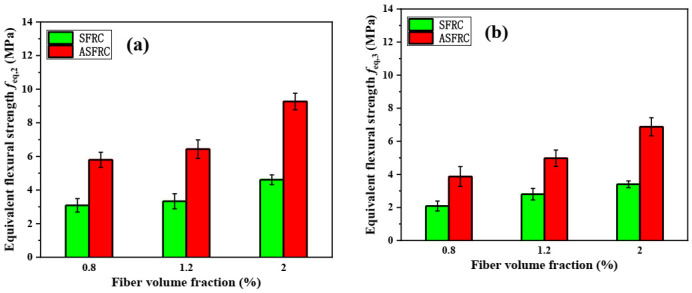
The equivalent flexure strength of SFRC and ASFRC specimens: (**a**) Equivalent flexure strength $f_{eq,2}$ and (**b**) Equivalent flexure strength $f_{eq,3}$.

The equivalent flexural strength ($f_{eq,2}$, $f_{eq,3}$) were calculated according to RILEM TC 162-TDF [38] and were shown in Figure 6. It can be seen in Figure 6 that, like the flexural strength and residual flexural strength, the equivalent flexural strengths $f_{eq,2}$ and $f_{eq,3}$ of ASFRC were also significantly higher than that of SFRC. Among them, $f_{eq,2}$ of ASFRC was 87.7%, 93.0%, and 101.0% higher than that of SFRC under 0.8%, 1.2%, and 2.0% of fiber volume content while $f_{eq,3}$ of ASFRC was 85.4%, 77.5%, and 102.3% higher than that of SFRC at various fiber volume contents.

**Figure 7:**
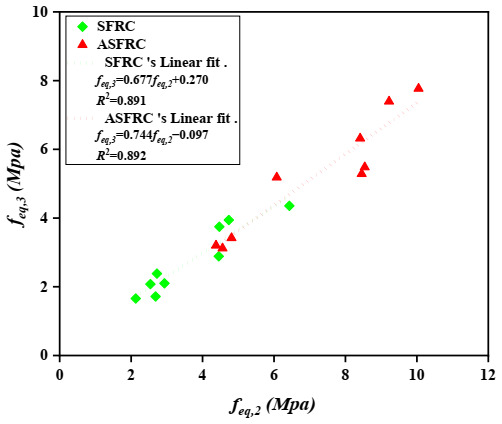
Relationship between equivalent flexural tensile strengths: $f_{eq,2}$ and $f_{eq,3}$.

**Figure 8:**
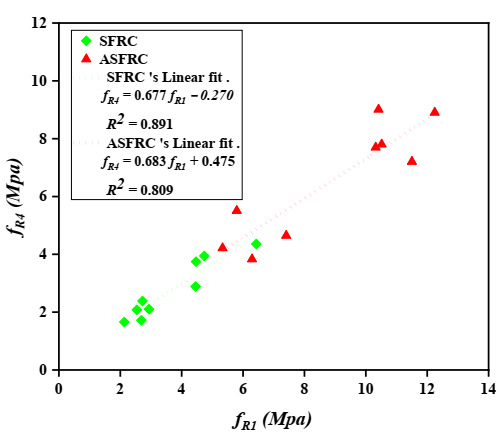
Relationship between residual flexural tensile strengths: $f_{R1}$ and $f_{R4}$.

Barros et al. [39] investigated the correlations between the post-peak parameters evaluated on concrete according to RILEM TC 162-TDF [38]. He suggested a linear relationship between the equivalent tensile strengths $f_{eq,2}$ and $f_{eq,3}$, in which $f_{eq,3}$ was about 92% of the $f_{eq,2}.$ A linear trend between $f_{eq,2}$ and $f_{eq,3}$ was also obtained and was presented in Figure 7. The value of $f_{eq,3}$ ranges from 67% to 74% of $f_{eq,2}$ for SFRC and ASFRC, respectively. Further considerations made by Barros et al. [39] also suggested a linear trend between $f_{R1}$ and $f_{R4}$ with $f_{R4}$ being at 93% of *f_R_*_,1_. Based on the research, the residual flexural tensile strength $f_{R4}$ of ASFRC is 68% and SFRC is 67% of $f_{R1}$ (see Figure 8). The value of $R^{2}$ achieved by Barros et al. [39] for SFRC was equal to 0.933 for $f_{eq,3}$ - $f_{eq,2}$ and 0.821 for $f_{R1}$ - $f_{R4}$ relationships. The scatter in the $f_{eq,3}$ - $f_{eq,2}$ and $f_{R1}$ - $f_{R4}$ relationships is clearly visible while analyzing the value of $R^{2}$, it ranges from 0.891 to 0.892 and 0.809 to 0.891 for the $f_{eq,3}$ - $f_{eq,2}$ and $f_{R1}$ - $f_{R4}$ relationships, respectively. Both sets of relationships characterizing ASFRC and SFRC are less steep than those created by Barros et al. [39]. Thus, the relationship between residual and equivalent flexural tensile strength is different than the one obtained by Barros, which should be further studied.

To prevent brittleness in a structural member, the fib Model code 2010 [40] suggests that steel fibers can be used to substitute conventional reinforcement at the ultimate limit state if two conditions are satisfied:

(i) $f_{R1K}/f_{LK}$ ≥ 0.4 and

(ii) $f_{R3K}/f_{LK}$ ≥ 0.5.

where $f_{LK}$ is the characteristic value of limit of proportionality determined using EN 14651-2005 [41]. *f*_R1K_ and *f*_R3K_ are the characteristic residual flexural strength of ASFRC and SFRC corresponding to the characteristic serviceability limit at CMOD_1_ and characteristic ultimate limit at CMOD_3_, respectively. The strength ratio $f_{R1K}/f_{R3K}$ represents the softening or hardening of the composite behavior. The characteristic values are calculated using the normal distribution, with a confidence interval of 0.75 [40]. Table 3 presents the characteristic values $f_{LK}$, $f_{R1K}$, and $f_{R3K}$ of all specimens and the ratios $f_{R1K}/f_{LK}$ and $f_{R3K}/f_{LK}$.

Comparing the serviceability limit expressed by $f_{R1K}/f_{LK}$ presented in Table 3, all SFRCs and ASFRCs composites satisfied the requirement to prevent brittle failure in the structural member for enabling conventional reinforcement substitution proposed by the fib model code 2010 [40] ($f_{R1K}/f_{LK}$ ≥ 0.4, and $f_{R3K}/f_{LK}$ ≥ 0.5). The results obtained for the $f_{R3K}/f_{LK}$ indicate the effectiveness of the fiber bridging up to the ultimate limit state at CMOD_3_. As a result, this recommendation was found to be very satisfactory in determining the post-peak parameters of SFRC with very low coefficients of variation. By analyzing the test results, it can be concluded that the orientation of steel fiber has a pronounced effect on post-peak parameters. Therefore, the alignment of steel fibers is an effective way to improve the post-peak parameters. Compared with SFRC, ASFRC was more effective in improving the post peak parameters for all tested fiber volume fractions. This can be attributed to the significantly improved reinforcement effect of aligned steel fibers.

### 3.4. Fracture Energy

The fracture energy is one of the basic parameters of advanced analyses which describes the capability of concrete to withstand the load, depending on its strain deformation characteristics. The fracture energy is defined as the amount of energy necessary to create a crack in the unit surface area projected in a plane parallel to the crack direction [42]. According to the RILEM [43], fracture energy can be calculated as:

(8) $$G_{F}=\frac{W_{0}+mg\delta }{b\left(d-a_{0}\right)}$$

where, $W_{0}$ is the area under the load–deflection curve (N·m), m is the mass of the specimen (kg), g is the acceleration of gravity (N/kg), δ is the deflection at final fracture(m), and $a_{0}$ is the notch depth (m).

Ideally, the fracture energy is calculated for the complete process of loading. This is typical for plain concrete but in the case of steel fiber reinforced composites the residual load carrying capacity usually remains for a long time in the post-peak phase of loading. Therefore, for steel fiber reinforced cementitious composites, only a certain area from the load–deflection curve is defined. In this study, the fracture energy was evaluated from the load–deflection curve up to 3 mm of deflection.

**Figure 9:**
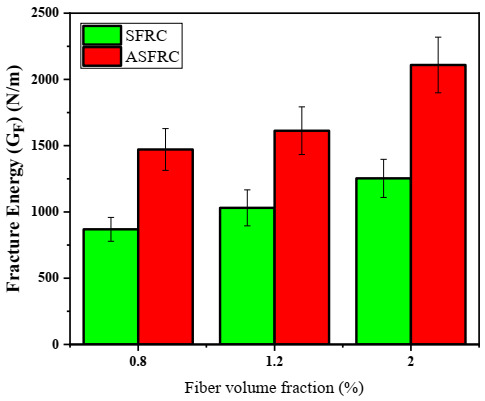
Average fracture energy of ASFRC and SFRC with respect to the steel fiber volume fractions.

The fracture energy obtained for SFRC and ASFRC is presented in Figure 9. It is clear that the fracture energy (*G_F_*) is significantly improved with the fiber orientation and incorporation for both SFRC and ASFRC [44,45]. The average fracture energy obtained for ASFRC was 1471.3 N/m, 1612.9 N/m, and 2109.3 N/m, while for SFRC it was found to be 868.5 N/m, 1030.9 N/m, and 1260.2 N/m with the fiber volume fraction of 0.8%, 1.2%, and 2.0%, respectively. It is found that the fracture energy of ASFRC was 56–70% higher than that of SFRC.

Iftikhar et al. [17] also investigated the fracture energy of SFRC and ASFRC. He found that the fracture energy of SFRC was about 3500–4500 N/m while the parameter of ASFRC was about 5500–8200 N/m, which was significantly higher than that in this study. This may be because of the difference in size of the specimen. Besides, the choice of deflection when calculating the area under the load–deflection curve also has a significant effect on the value of fracture energy. However, the conclusion that the fracture energy of ASFRC was higher than that of SFRC was widely accepted. Similar conclusions were also reported by González [46] and Antonio [47]. On the other hand, due to the use of straight steel fiber, the fracture energy of ASFRC was 31–70% greater than SFRC [17]. Comparing with the 56–70% increase in this study, it can be concluded that the use of hooked-end steel fiber can further improve the fractural properties.

### 3.5. Fiber Orientation Efficiency Factor and Reinforcement Mechanism

The number of steel fibers belonging to each angle zone was recorded and is presented in Table 4. It can be seen from Table 4 that compared with the SFRC specimens, the ASFRC specimens have much more fibers in the 0–15° angle range than in any other angle ranges. Steel fibers having 0–15° angle range are most favorable since these fibers are the best aligned with the direction of the tensile load. Moreover, with the same fiber addition, the total number of fiber for ASFRC was larger than that of SFRC. The reason is that some fibers in SFRC specimens cannot cross the cracked surface due to their directions being parallel to the surface, even close to the section. However, the fibers in ASFRC are always intersected with the cracked surface because of the alignment of steel fibers [48].

Table 4 also gives the fiber orientation efficiency factor according to Equation (5). The orientation efficiency factors ($\eta_{\theta }$) for the ASFRC specimens with steel fiber volume fractions of 0.8%, 1.2%, and 2.0% were 0.71, 0.85, and 0.81, respectively, while the corresponding values for SFRC specimens were 0.59, 0.59, and 0.58 for 0.8%, 1.2%, and 2.0%, respectively. Therefore, the fiber orientation efficiency factors were about 0.80 in the ASFRC specimen, while it was about 0.58 in the SFRC specimen. The results indicate that a higher value of orientation efficiency factors ($\eta_{\theta }$) of all ASFRC specimens than that of the SFRC specimens, which means more steel fibers are aligned along the direction of tensile stress in specimens. Therefore, when the volume fraction of steel fibers is the same, there are more fibers playing a role in reinforcement in ASFRC specimens due to the larger total numbers and the higher fiber orientation efficiency factor. Therefore, the mechanical properties of ASFRC were improved significantly.

## 4. Conclusions

This article analyzed the key parameters of preparing aligned hooked-end steel fiber cementitious composites (ASFRC). Besides, the three points bending (TPB) test was carried out to investigate the fractural properties of ASFRC. Based on the test results, the following conclusions can be drawn:

1. The strength of SFRC and ASFRC structure is determined by the fiber orientation and fiber distribution, which remarkably depends on the rheology of mixture and magnetic induction of the electromagnetic field. In order to prepare ASFRC, the appropriate level of viscosity of the mixture and the magnetic induction were about 10–300 Pa·s and 0.15 × 10^−4^–2.0 × 10^−4^ T, respectively.

2. The effect of fiber orientation was found to be very small on pre-cracking behavior but significant on post-cracking behavior. The alignment of steel fiber contributed to the higher ultimate load and post-cracking stage. Compared with SFRC, the cracking load increased about 24–55% and the ultimate load increased about 51–86% for ASFRC depending on the fiber addition content.

3. The load–CMOD curve showed that under the same CMOD, the ASFRC specimens exhibited superior load-bearing capacity. When the CMOD of specimens exceeded about 0.2 mm, the load of the ASFRC specimen was still growing while the load of the SFRC specimen began to fall after the initial crack of the matrix.

4. In terms of energy consumption, the flexural tensile strength and the residual flexural strength of ASFRC were found to increase up to 105% and 100%, respectively, compared with those of SFRC. On the other hand, the fracture energy of ASFRC was 69.4%, 56.5%, and 67.4% higher than that of SFRC and the reinforcement effect of hooked-end steel fiber was higher than straight steel fiber. The residual flexural strengths $f_{R1}$ and $f_{R4}$ were also about 85–100% higher than those of SFRC and were found to confirm with the RILEM TC162–TDF.

5. The number of fibers in the fracture surface of ASFRC was higher than that of SFRC and the orientation efficiency factor of ASFRC and SFRC was 0.8 and 0.58, respectively. Due to the alignment of fibers in ASFRC specimens, there are more fibers playing a role in the reinforcement effect, which explains the higher fracture properties of ASFRC.

## Figures and Tables

**Table 1:** Mix proportions of steel fiber reinforced cement-based composites.

Specimen ID	w/c	Water $\left(kg/m^{3}\right)$	Cement $\left(kg/m^{3}\right)$	Sand $\left(kg/m^{3}\right)$	Steel Fibers $\left(kg/m^{3}\right)$
ASFRC-0.8%	0.36	235	653	1306	62.4
SFRC-0.8%	0.36	235	653	1306	62.4
ASFRC-1.2%	0.36	236	655	1310	93.6
SFRC-1.2%	0.36	236	655	1310	93.6
ASFRC-2.0%	0.36	238	661	1322	156.0
SFRC-2.0%	0.36	238	661	1322	156.0

**Table 2:** Properties and configuration of hooked-end steel fibers.

Fiber Type	Length (mm)	Diameter (mm)	Aspect Ratio	Tensile Strength (MPa)	Elastic Modulus (GPa)	Fiber Configuration
Hooked-end steel fiber	25	0.50	50	1250	200	** 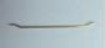 **

**Table 3:** Summary of the characteristic values of all specimens.

Specimen ID	$f_{LK}$ (MPa)	$f_{R1K}$ (MPa)	$f_{R3K}$ (MPa)	$f_{R1K}/f_{LK}$	$f_{R3K}/f_{LK}$
ASFRC 0.8%	4.452	4.249	3.173	0.954	0.746
SFRC 0.8%	4.178	2.329	1.879	0.557	0.806
ASFRC1.2%	4.566	5.370	5.002	1.175	0.931
SFRC 1.2%	4.501	2.874	2.181	0.638	0.758
ASFRC 2%	6.376	8.862	9.272	1.389	1.046
SFRC 2%	4.597	3.614	2.820	0.786	0.780

**Table 4:** Number of steel fibers in fractured cement-based composite sections.

Specimen ID	Numbers				Total numbers	$\left(\eta_{\theta }\right)$
	0–15°	15–45°	45–75°	75–90°		
ASFRC-0.8%	127	91	26	15	259	0.71
SFRC-0.8%	56	85	45	19	205	0.59
ASFRC-1.2%	292	67	32	9	400	0.85
SFRC-1.2%	83	127	82	25	317	0.59
ASFRC-2%	407	160	39	13	619	0.81
SFRC-2%	106	174	120	34	434	0.58

## Data Availability

Available on request from the corresponding author.

## Nomenclature

W	Weight of steel fiber or gravity
$F_{b}$	Buoyant force subjected to steel fiber
$F_{m}$	Magnetic force subjected to steel fiber
$F_{d}$	Viscous resistance subjected to steel fiber
$C_{D}$	Drag coefficient
A	The projected area of the fiber on the plane
	vertical to the direction of rotation
$l_{f}$	Length of steel fiber
ρ	Density of mixture
v	Relative speed between fiber and matrix
$A_{f}$	Cross-section area of steel fiber
B	Magnetic induction
μ	Magnetic permeability of steel fiber
$\mu_{0}$	Vacuum permeability
I	Current of the solenoid
n	The number of solenoid turns
l	The length of solenoid
S	The span of beam
$a_{0}$	The notch depth
d	The height of beam
$\eta_{\theta }$	Average orientation efficiency factor of steel fiber
N	Total number of fibers in the cracked section
$\theta_{i}$	The angle between the steel fiber and the axis of the specimen
$f_{L}$	Flexural tensile strength
$F_{L}$	The maximum load for CMOD ≤ 0.05 mm
b	Width of beam
$h_{sp}$	Effective height of beam
$f_{Rj}$	Residual flexural strength
$f_{eq,2}$,$f_{eq,3}$	Equivalent flexural strength
$G_{F}$	Fracture energy
$W_{0}$	The area under the load–deflection curve
m	The mass of the specimen
g	Acceleration of gravity
δ	The deflection at final fracture
